# Supplementary figures and images for: Spatial distribution of cytoskeleton-mediated feedback controls cell polarization: A computational study
Source: PLoS Comput Biol. 2025 Oct 9;21(10):e1013036. doi: 10.1371/journal.pcbi.1013036 (PMC12539730; doi:10.1371/journal.pcbi.1013036)

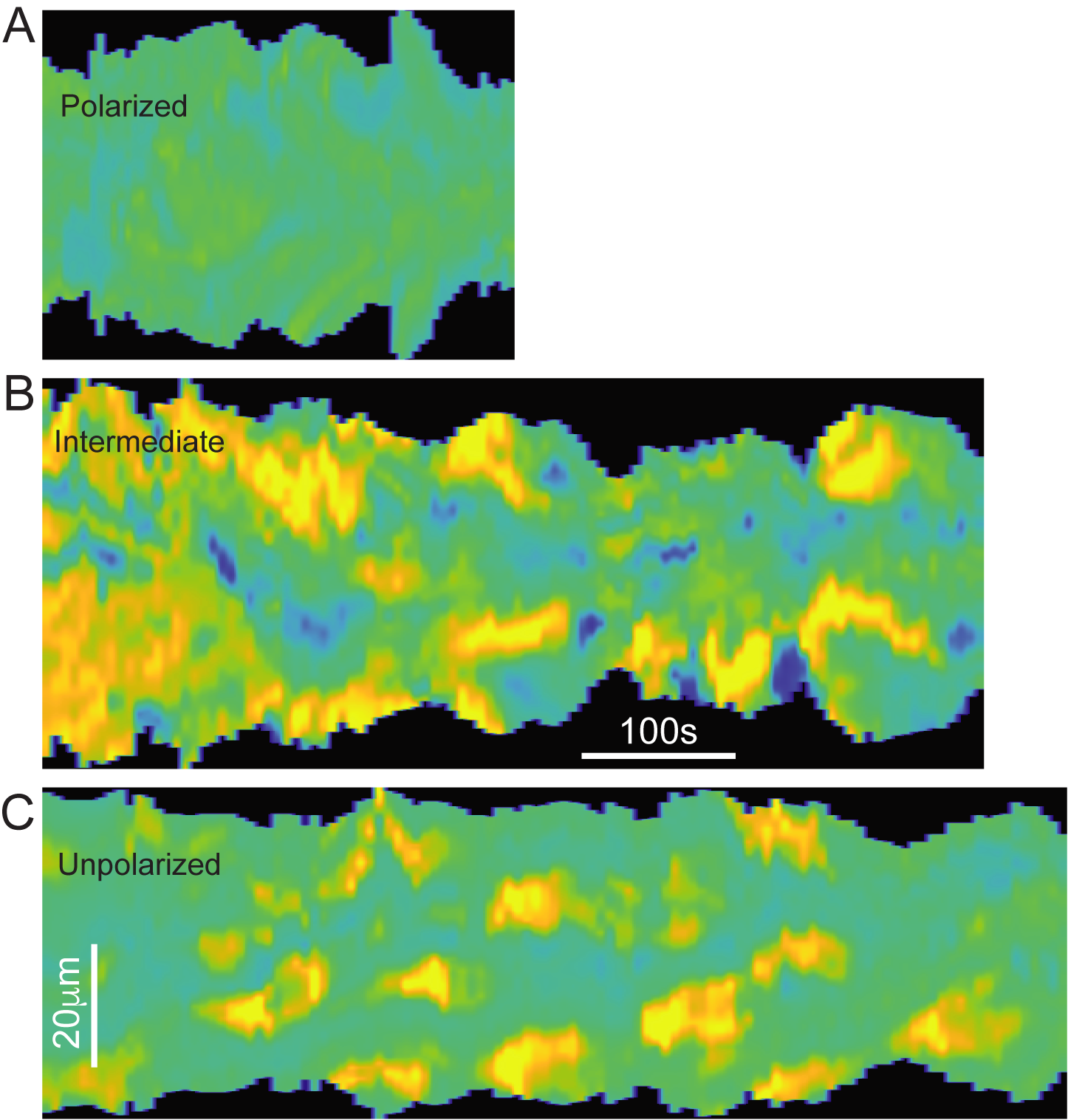

Supplement: S1 Fig — Kymographs showing the normalized membrane localization of RasGTP (RBD-GFP) in unpolarized (A), intermediate polarized (B), and fully polarized (C) cells. All kymographs share the same time scale on the x-axis and length scale on the y-axis. (TIFF) [file pcbi.1013036.s001.tif]

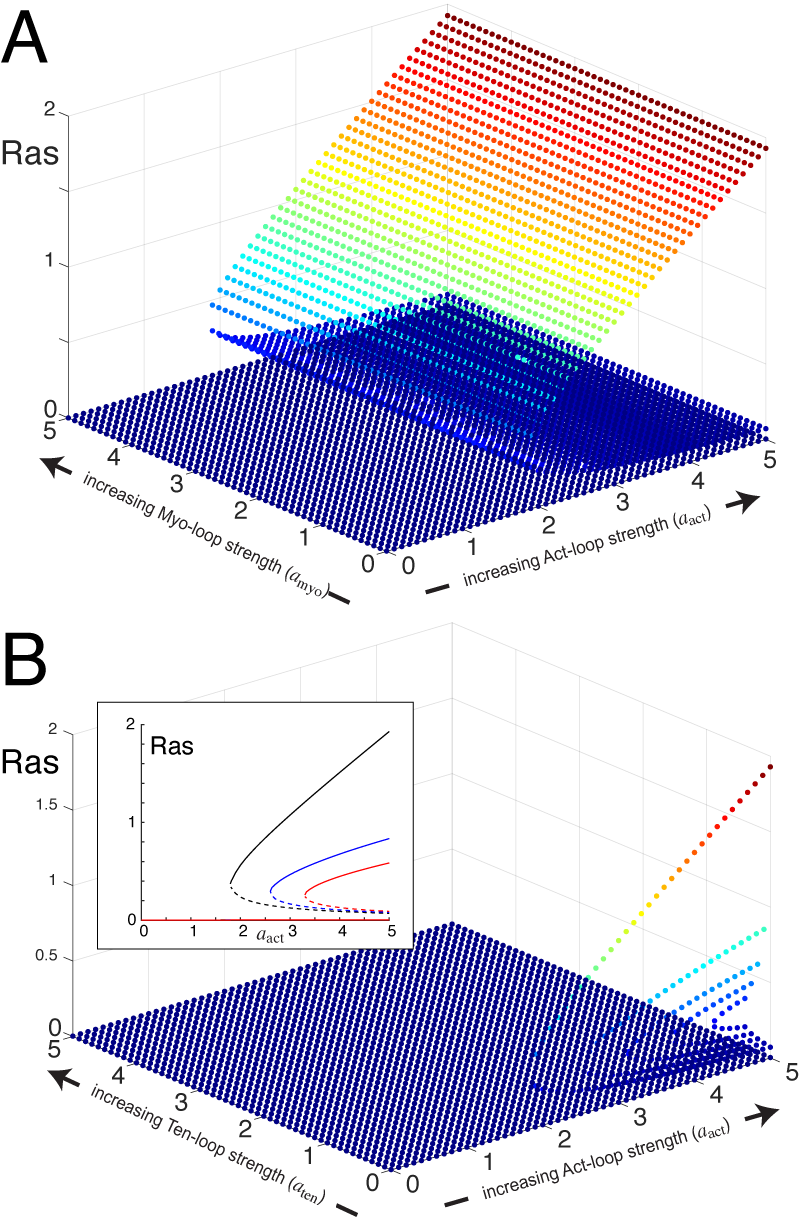

Supplement: S2 Fig — Each dot represents the Ras equilibrium value assuming no diffusion or noise as a function of the positive (aact) and negative (amyo/aten) feedback strengths. Both models show bistability, but that of global inhibition does so only for a restricted number of negative feedback strengths. The inset in panel B shows bistability and the equilibrium for these values. (TIFF) [file pcbi.1013036.s002.tif]

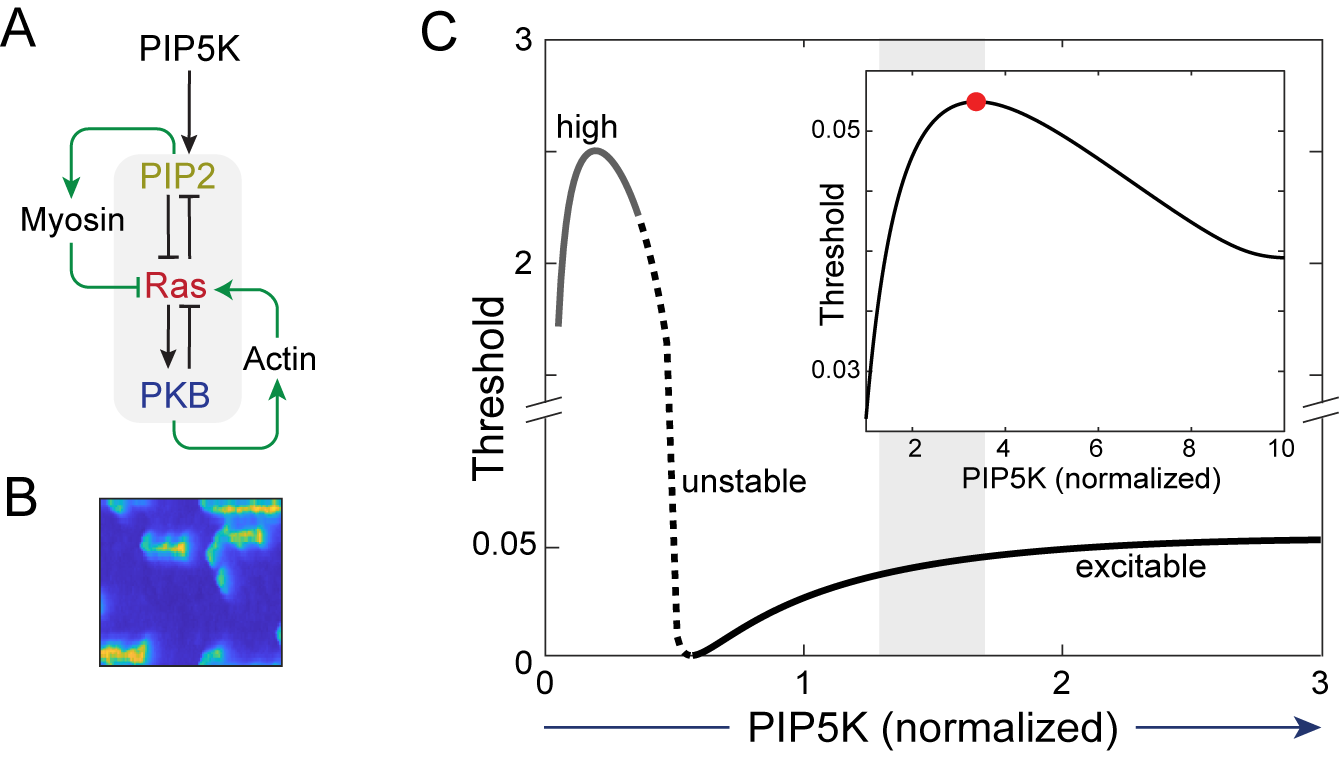

Supplement: S4 Fig — A. Schematic of a system in which varying levels of PIP5K alter the production rate of PIP2 in STEN (parameter b1 in Eq 2). The level PIP5K was set so that the production rate matched the basal level. B. Sample kymograph for this system when PIP5K=1. C. Effect on the excitable system activation threshold when the production level was changed by varying PIP5K levels. Around PIP5K, the system is excitable and shows an increase in the threshold (the system is harder to trigger) as PIP5K increases. There is some biphasic behavior, with the threshold peaking around PIP5K≈4 (inset). Lowering PIP5K levels makes the system easier to trigger (lowering threshold) and eventually goes unstable at around PIP5K≈0.6, marked by the dotted line. Eventually, the system reaches a very high level of activity, denoted by the solid, gray line, at PIP5K≈0.5. The shaded region denotes the range of PIP5K levels that was observed in simulations; in this range, the threshold shows a monotonic dependence on PIP5K levels. (TIFF) [file pcbi.1013036.s004.tif]

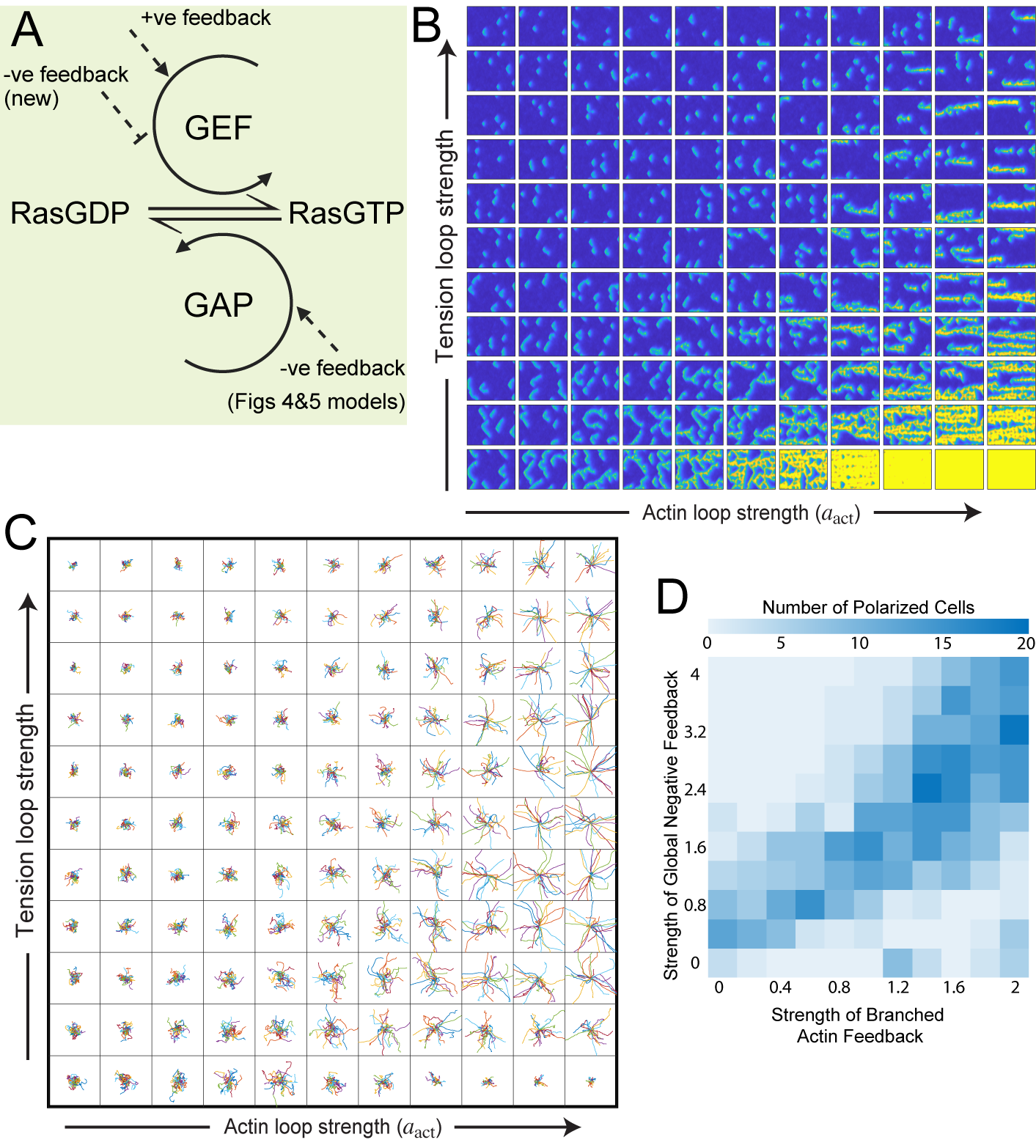

Supplement: S5 Fig — A. Schematic illustrating how positive and negative feedback influence the signaling pathway by modulating the degradation or production rate of Ras in different models. In the simulations of Fig 4 and Fig 5, the negative feedback is effected by increasing RasGTP hydrolysis of the RasGAP (Eq 6 and Eq 7, respectively.) In the simulations of this figure, the inhibition is achieved by reducing the guanine exchange by lowering the RasGEF action (Eq 8). All models used the same positive feedback which increases guanine exchange (Eq 5). B. Kymographs from simulations showing the effect of branched-actin-mediated positive feedback (ranging from 0 to 2 in steps of 0.2) and global negative feedback (ranging from 0 to 4.0 in steps of 0.4). C. Trajectories of simulated cells under varying feedback strengths, with 20 simulations per condition. Feedback strengths correspond to those in panel B. D. Heatmap showing the number of polarized cells in a population of 20 for each feedback strength condition represented in panels B and C. (TIFF) [file pcbi.1013036.s005.tif]
